# Supplementary material for: 7p22.2 Microduplication: A Pathogenic CNV?
Source: Genes (Basel). 2023 Jun 19;14(6):1292. doi: 10.3390/genes14061292 (PMC10297997; doi:10.3390/genes14061292)
Supplement: Supplementary file 1 [file genes-14-01292-s001.zip › genes-2440309-supplementary.pdf]

Supplementary Table S1. Primers for Real-Time PCR assays and relevant genetic regions under investigation

Patient	Localization	Forward Sequence	Backward Sequence
1	CHR12: 56670637-57016306	GGCCTGGGTGTTCAGTTCAA	AAACCCGATGTCTCCAATGG
1	CHR7: 3074785-3445950	GGGCTGGTCTGTACCTGAGTCT	CTGGATGTCACTCACCGGAAA
2	CHR7: 2967525-3422668		
